# Magneto-Optical Characteristics of Streptavidin-Coated Fe_3_O_4_@Au Core-Shell Nanoparticles for Potential Applications on Biomedical Assays

**DOI:** 10.1038/s41598-019-52773-7

**Published:** 2019-11-11

**Authors:** Chin-Wei Lin, Jian-Ming Chen, You-Jun Lin, Ling-Wei Chao, Sin-Yi Wei, Chiu-Hsien Wu, Chien-Chung Jeng, Li-Min Wang, Kuen-Lin Chen

**Affiliations:** 1 Graduate institute of applied physics, National Taiwan University, Taipei, Taiwan; 2 Institute of Nanoscience, National Chung Hsing University, Taichung, Taiwan; 3 Department of Physics, National Chung Hsing University, Taichung, Taiwan

**Keywords:** Nanoscience and technology, Biomedical engineering

## Abstract

Recently, gold-coated magnetic nanoparticles have drawn the interest of researchers due to their unique magneto-plasmonic characteristics. Previous research has found that the magneto-optical Faraday effect of gold-coated magnetic nanoparticles can be effectively enhanced because of the surface plasmon resonance of the gold shell. Furthermore, gold-coated magnetic nanoparticles are ideal for biomedical applications because of their high stability and biocompatibility. In this work, we synthesized Fe_3_O_4_@Au core-shell nanoparticles and coated streptavidin (STA) on the surface. Streptavidin is a protein which can selectively bind to biotin with a strong affinity. STA is widely used in biotechnology research including enzyme-linked immunosorbent assay (ELISA), time-resolved immunofluorescence (TRFIA), biosensors, and targeted pharmaceuticals. The Faraday magneto-optical characteristics of the biofunctionalized Fe_3_O_4_@Au nanoparticles were measured and studied. We showed that the streptavidin-coated Fe_3_O_4_@Au nanoparticles still possessed the enhanced magneto-optical Faraday effect. As a result, the possibility of using biofunctionalized Fe_3_O_4_@Au nanoparticles for magneto-optical biomedical assays should be explored.

## Introduction

Over the past few decades, nanotechnology has advanced rapidly. The special property of unique size of nanoparticles provides many advantages. Magnetic nanoparticles (MNPs) have been developed in many fields, such as biomedicine^1–5^, nano fluids^6^, magnetic resonance imaging^7–9^, and optics^10^. MNPs have recently attracted more attention for biomedical applications because of their magnetic and optical characteristics. MNPs can serve as drug carriers^11^, biosensors^12,13^, gene delivery systems^14^, etc.^15^.

Iron oxide MNPs are the most commonly used MNPs due to their superparamagnetic stability and biocompatibility^16^. The properties of Fe_3_O_4_ MNPs, such as size and shape, can be altered with different synthesis methods^17^ and as a result the MNPs can be specialized for different applications. Several synthesis routes have been reported including thermal decomposition^18^, co-precipitation^19^, and hydrothermal synthesis^20^. Each technique fits the demands of different biomedicine applications. Generally, the particle size of Fe_3_O_4_ MNPs is affected by pH variation^21^, temperature^22^, and stirring rate^23^ during the synthesis process.

To increase the stability and biocompatibility, the surface of the MNP generally needs to be modified with some noble metal or polymer. Several methods to modify MNPs made of iron oxide have been reported^3,4,24,25^. Gold, a noble metal with good biocompatibility, is commonly used in biomedical applications. Due to their biocompatibility gold coated MNPs have been developed and widely studied^26,27^. Moreover, gold coated MNPs simultaneously possess magnetic and plasmonic characteristics. Jain *et al*. reported that the magneto-optical Faraday effect in gold-coated iron oxide nanocrystals was enhanced due to surface plasmon resonance enhanced magneto-optics (SuPREMO)^28^. However, the nanoparticle surface generally needs to be modified with biomaterials or proteins for applications in biomedicine. It is well known that surface plasmon resonance (SPR) is very sensitive to the surface state of the nanoparticle. The SPR of a nanoparticle is highly responsive to small changes in the local refraction index^29^. Hence, the surface modification of biomaterials certainly alters the characteristics of the SPR.

In this work, we synthesized Fe_3_O_4_@Au core/shell magnetic nanoparticles and coated their surface with streptavidin (STA) to study how the addition of STA impacted the magneto-optical Faraday effect. STA is a widely used biomaterial for developing new biomedical methods because the conjugation of STA and biotin is very strong. It is commonly used to investigate the quantification process with biotin. We experimentally demonstrated that Fe_3_O_4_@Au core-shell MNPs were still able to enhance the magneto-optical Faraday rotation even after surface modification with STA. This result suggests that SuPREMO is a promising effect to exploit in biomedical assay techniques based on the magneto-optical effect, such as the Faraday immunoassay system^30^.

In our previous work^30^, we have demonstrated that the Faraday magneto-optical measurement with biofunctionalized magnetic nanoparticles (BMNs) results in a simple, convenient, and sensitive tool for assaying biomarkers. Due to the antibody–antigen interactions, BMNs conjugated with the biotargets to form large magnetic clusters over time. The magnetic characteristics of the BMNs reagent are altered as well. The Faraday rotation angle varies as a function of the size of the MNP. Therefore, we aim to observe the clustering process by measuring the Faraday effect of MNPs. Since SuPRMO MNPs possess the special characteristic of Faraday rotation enhancement, biofunctionalized SuPRMO MNPs are a potential reagent for increasing the sensitivity of the magneto-optical Faraday immunoassay technique.

## Results and Discussions

### X-ray diffraction (XRD) & UV-Vis spectrum

**Figure 1:**
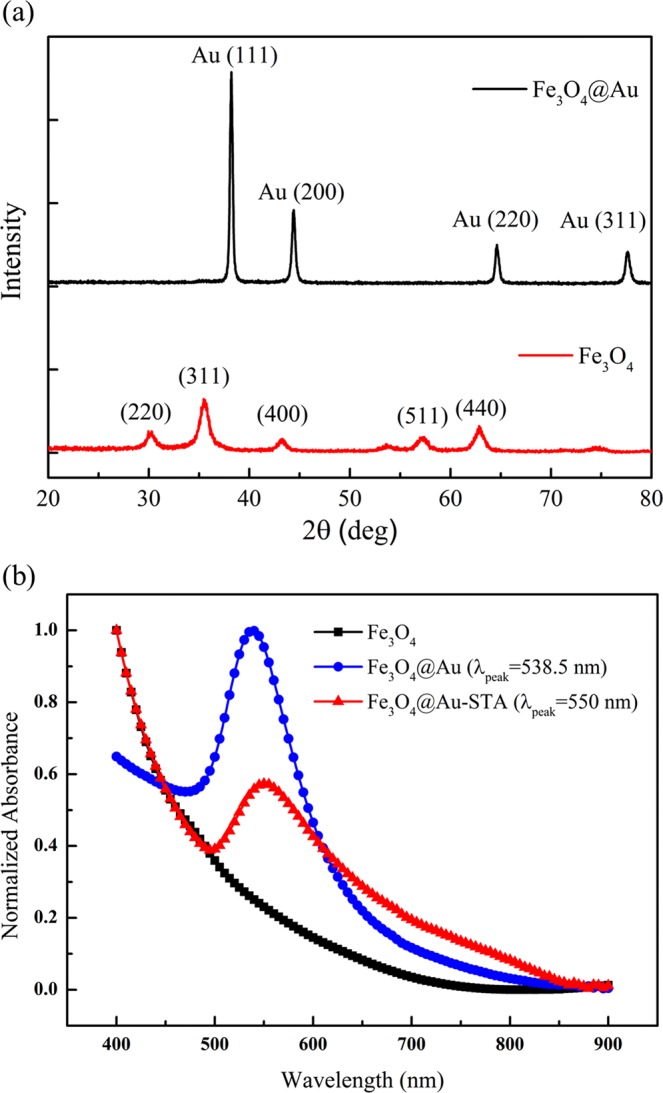
(**a**) Powder XRD patterns and (**b**) UV-Vis spectra of the Fe_3_O_4_, Fe_3_O_4_@Au core-shell and Fe_3_O_4_@Au-STA MNPs.

Figure 1a shows the powder X-ray diffraction (XRD) patterns of Fe_3_O_4_ and Fe_3_O_4_@Au core-shell MNPs. The diffraction angle of the (311) peak of the raw MNPs occurs at 35.46°, which means that the composition of the MNPs is magnetite before reducing the Au shell^31^. The XRD data showed that the synthesized particles are Fe_3_O_4_ with good crystallinity. After coating the MNPS with an Au shell, the XRD signals of the Fe_3_O_4_ core were shielded by the gold layer because of the heavy atom effect^24^. The absorbance of the synthesized particles was measured using ultraviolet-visible spectroscopy (UV-Vis) (U-2800A, HITACHI). The UV-Vis spectra (Fig. 1b) shows that the absorbance of pure Fe_3_O_4_ MNPs monotonically decreased with the wavelength of light. However, the Fe_3_O_4_@Au core-shell MNPs exhibited an absorption peak at a wavelength of approximately 538.5 nm due to the SPR effect of the gold layer. After the bonding of STA, the wavelength of the absorption peak of the Fe_3_O_4_@Au-STA MNPs increased to around 550 nm. The result clearly shows that the STA modification induces a red shift of the UV-Vis spectrum. This means that the refractive index of the STA does indeed alter the SPR condition of the Fe_3_O_4_@Au MNPs.

### Dynamic light scattering

**Figure 2:**
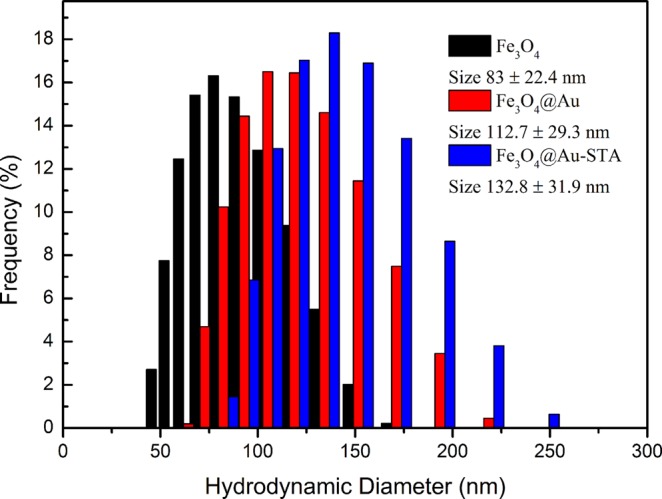
Distribution of the hydrodynamic diameter of the Fe_3_O_4_, Fe_3_O_4_@Au core-shell and Fe_3_O_4_@Au-STA MNPs.

Figure 2 shows the hydrodynamic sizes of the Fe_3_O_4_, Fe_3_O_4_@Au core-shell, and Fe_3_O_4_@Au-STA MNPs measured by dynamic light scattering (DLS) (SZ-100Z, HORIBA). Table 1 shows the hydrodynamic diameter, polydispersity index (PI), and Zeta potential of the Fe_3_O_4_, Fe_3_O_4_@Au, and Fe_3_O_4_@Au-STA MNPs. The average hydrodynamic diameters of Fe_3_O_4_, Fe_3_O_4_@Au, and Fe_3_O_4_@Au-STA MNPs are 83.0 ± 22.4, 112.7 ± 29.3, and 132.8 ± 31.9 nm, respectively. As expected, the mean size and dispersion of the distribution of the MNPs increases as the amount of covering material increases. The Zeta potential data show that the Fe_3_O_4_, Fe_3_O_4_@Au, and Fe_3_O_4_@Au-STA are both stable in the solution.

**Table 1:** Hydrodynamic diameter, polydispersity index (PI) and Zeta potential of the Fe_3_O_4_, Fe_3_O_4_@Au, and Fe_3_O_4_@Au-STA MNPs.

MNPs	Size(nm)	PI	Zeta Potential(mV)
Fe_3_O_4_	83.0 ± 22.4	0.258	−66.1
Fe_3_O_4_@Au	112.7 ± 29.3	0.300	−57.8
Fe_3_O_4_@Au-STA	132.8 ± 31.9	0.338	−46.8

### High-resolution transmission electron microscopy (HRTEM)

**Figure 3:**
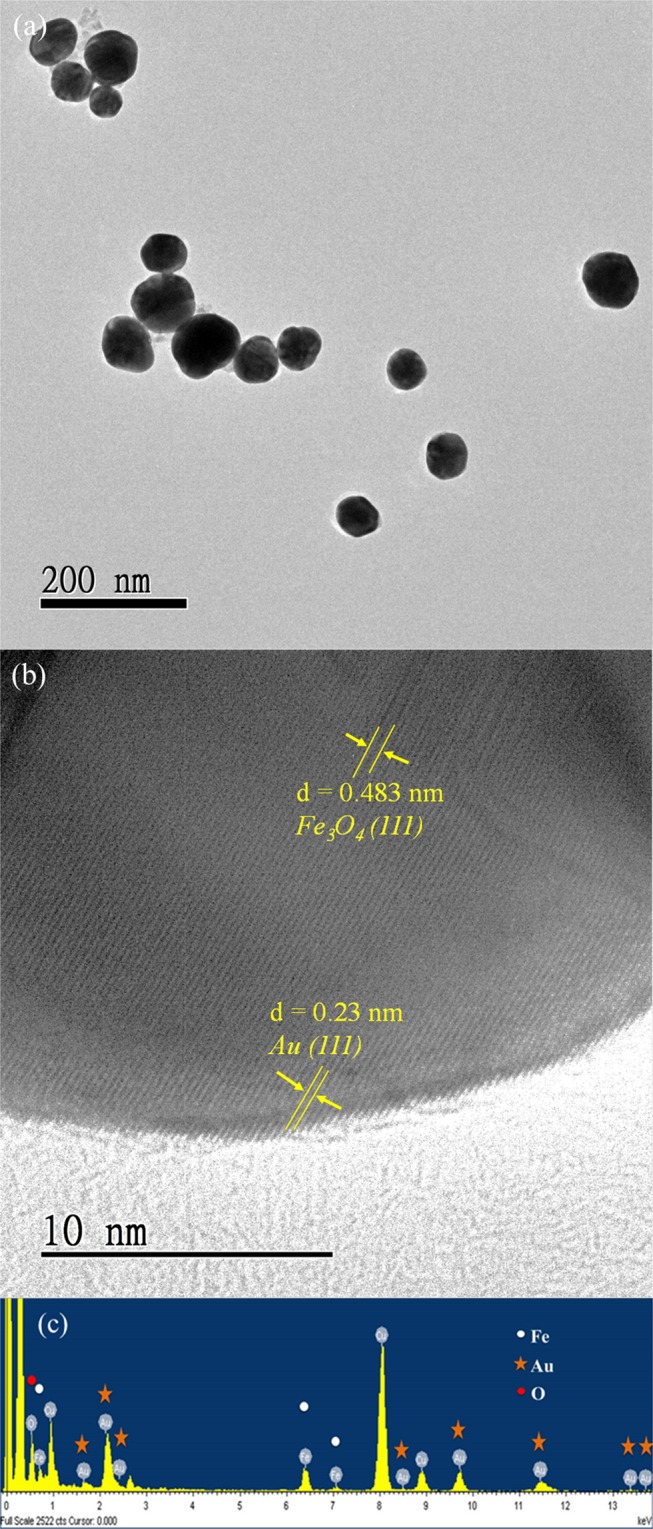
(**a**) TEM images of the Fe_3_O_4_@Au MNPs. The scale bar is 200 nm. (**b**) Magnified image of the gold shell on the Fe_3_O_4_ core. The scale bar is 10 nm. (**c**) EDS spectrum of the Fe_3_O_4_@Au MNPs.

Figure 3a presents a high-resolution transmission electron microscopy (HRTEM) (JEM-2010, JEOL Co. Ltd) image of the Fe_3_O_4_@Au core-shell MNPs. It shows that the shape of the synthesized MNPs was approximately spherical and their average size was about 66 nm. Figure 3b shows a magnified HRTEM image near the surface of a Fe_3_O_4_@Au core-shell MNP. The crystal structure of the gold shell on the Fe_3_O_4_ core can be clearly seen. The TEM image simultaneously shows the Au lattice near the particle surface and the Fe_3_O_4_ lattice at the core. The Fe_3_O_4_ lattice is relatively blurry because the electron beam has difficulty penetrating to the center of particle. The analysis of the energy-dispersive X-ray spectroscopy (EDS) (JEM-2010, JEOL Co. Ltd) for the Fe_3_O_4_@Au core-shell MNPs clearly revealed that the synthesized particles contained the elements Fe, O, and Au (Fig. 3c). The results of characteristic analysis proved that the Fe_3_O_4_@Au core-shell MNPs were successfully synthesized.

### Magnetization curve

**Figure 4:**
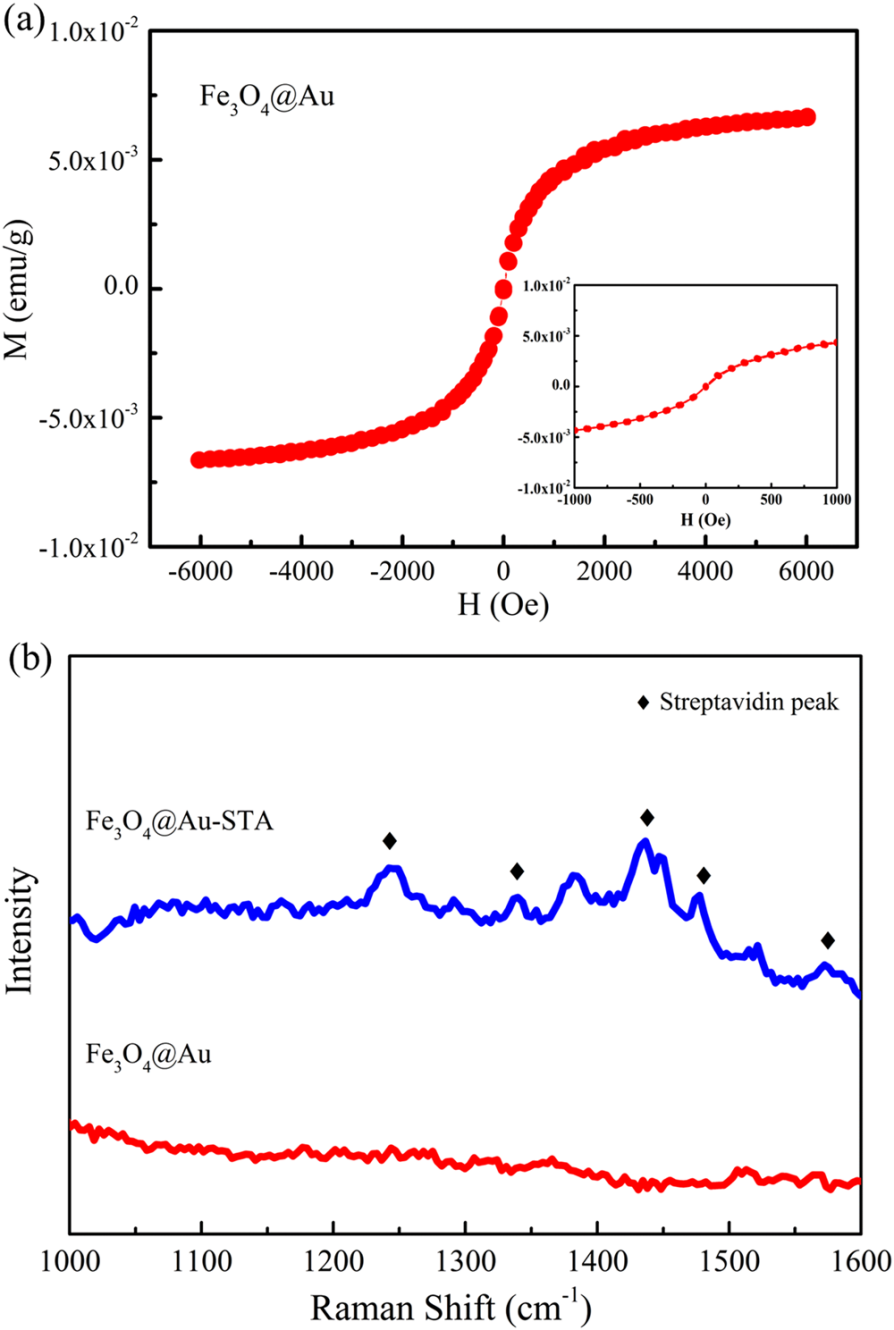
(**a**) Magnetic hysteresis curve of the Fe_3_O_4_@Au MNPs. The inset shows the magnetization of the Fe_3_O_4_@Au MNPs under a small magnetic field. (**b**) Raman spectra of the Fe_3_O_4_@Au MNPs before and after coating STA. The markers indicate the characteristic Raman peaks of STA.

Figure 4a shows the magnetization curve of the Fe_3_O_4_@Au MNP reagent measured by a SQUID magnetic property measurement system (MPMS, Quantum Design, Inc) at 300 K. The inset in Fig. 4a shows that there was no hysteresis in the magnetization curve even under a small magnetic field. Fe_3_O_4_@Au MNPs exhibited the characteristics of superparamagnetic material; noting that the magnetization shown is the magnetization of the MNP reagent, not the magnetization of the MNP powder. A 3-D Nanometer Scale Raman PL Microspectrometer (Tokyo Instruments, INC.) was used to determine whether STA was successfully coated on the surface of Fe_3_O_4_@Au MNPs. Figure 4b shows the Raman spectra of the Fe_3_O_4_@Au MNPs before and after coating STA. After STA was coated, peaks emerged in the region of 1200–1600 cm^−1^ with respect to the Raman signals of Fe_3_O_4_@Au MNPs. The marked peaks in Fig. 4b showed the presence of STA. Peaks at 1254 and 1279 cm^−1^ represent the amide III region and the peak at 1447 cm^−1^ represents the δ-CH_2_ and δ-CH_3_ bands. The Trp10, Trp7, Trp5, and Trp2 signals are at 1243, 1341, 1461, and 1580 cm^−1^, respectively^32^. All these peaks are the characteristic Raman signals of STA^32^ indicating that STA was successfully coated on the surface of the Fe_3_O_4_@Au MNPs.

### Faraday rotation measurement

**Figure 5:**
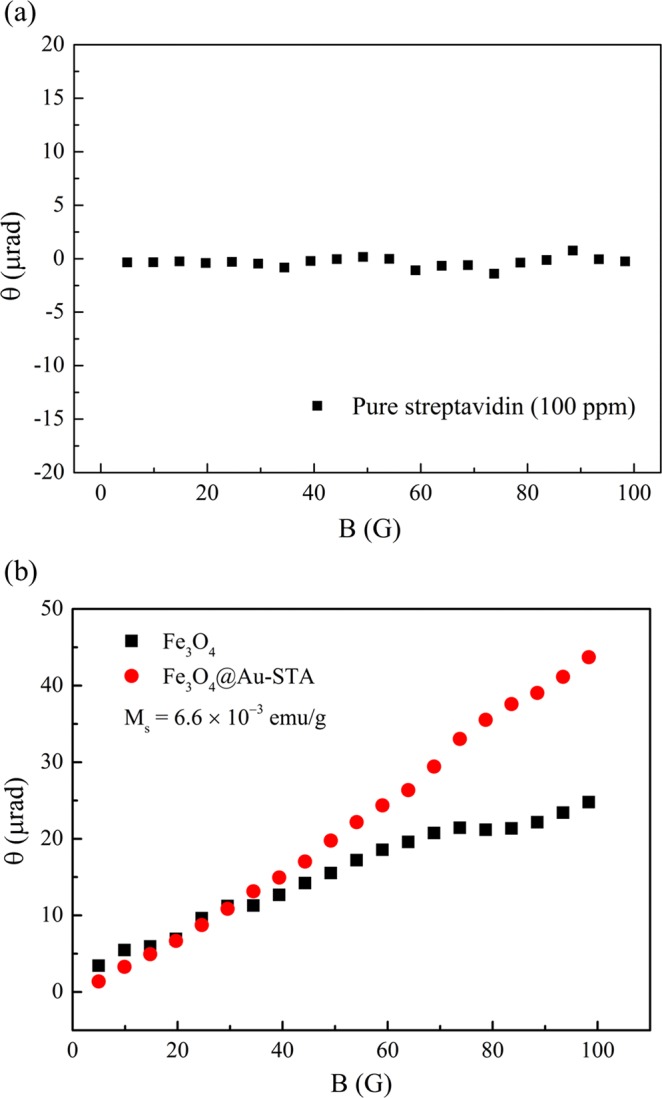
(**a**) Faraday rotation spectrum of the pure STA reagent with the concentration of 100 μg/mL. (**b**) Faraday rotation spectra of Fe_3_O_4_@Au-STA and Fe_3_O_4_ MNPs. The saturation magnetizations of the Fe_3_O_4_@Au-STA and Fe_3_O_4_ MNPs reagents both were 6.6 × 10^−3^ emu/g.

To confirm the Faraday rotation enhancement of the Fe_3_O_4_@Au-STA MNPs, we checked the magneto-optical characteristic of the pure STA reagent. Figure 5a is the Faraday rotation spectrum of pure STA reagent (100 μg/mL) as a function of the applied magnetic field. Clearly the magneto-optical Faraday effect of the pure STA reagent was extremely weak when the applied magnetic field was less than 100 gauss. Recalling that Fig. 1b revealed that the STA modification does indeed alter the SPR condition of the Fe_3_O_4_@Au NPs; Fig. 5b shows the Faraday rotation spectra of the Fe_3_O_4_@Au-STA and Fe_3_O_4_ MNPs reagents as a function of the applied magnetic field. To exclude the influence of magnetization on the Faraday rotations, the saturation magnetizations of the measured samples were controlled (M_s_ = 6.6 × 10^−3^ emu/g for both). Figure 5b shows that the Faraday rotation of the Fe_3_O_4_@Au-STA was larger than that of Fe_3_O_4_ for an applied magnetic field larger than 30 gauss. The gold layer of core shield MNPs can be seen as an optical cavity with multiple resonance modes. When the light at a corresponding frequency illuminates the cavity, the energy of that light is stored inside the cavity. The result is that the MNP inside cavity senses a stronger electromagnetic field than the MNP without a cavity. The enhanced interaction between the MNPs and light results in the larger Faraday rotation. This result proves that the Fe_3_O_4_@Au-STA MNPs still possessed the SuPREMO effect which enhances the Faraday rotation even after the STA coating was applied. The biomaterial modified magneto-plasmonic nanoparticle is promising for applications based on the magneto-optical Faraday effect. In our previous work^30^, we successfully developed a Faraday immunoassay technique based on the magneto-optical Faraday effect and biofunctionalized MNPs. Now, these experimental results suggest that biofunctionalized magneto-plasmonic nanoparticle can be exploited to improve sensitivity using the Faraday immunoassay technique.

In summary, we synthesized the Fe_3_O_4_@Au core-shell MNPs and coated particle surfaces with STA. We observed that the Fe_3_O_4_@Au-STA MNPs still possess the Faraday rotation enhancement after conjugating the biomaterial on the surface of the gold layer. The experimental results imply that the biofunctionalized Fe_3_O_4_@Au core-shell MNPs still had the effect of SuPREMO and are promising for magneto-optical biomedical applications.

### Methods Synthesis of the bio-functionalized core-shell Fe_3_O_4_@Au nanoparticles

In this study, iron oxide nanoparticles were prepared by co-precipitation of Fe(II) and Fe(III) first. An iron salt aqueous solution was combined with Ferric chloride (FeCl_3_.6H_2_O) and ferrous chloride (FeCl_2_.4H_2_O) at a ratio of 2:1. The iron salt aqueous solution was heated to 80 °C. Then, 28% NH_4_OH (W/V) was added to the iron salt aqueous solution and stirred for 30 min to form Fe_3_O_4_ nanoparticles. After that, Fe_3_O_4_ nanoparticles were obtained by magnetic separation. DI water was used to wash the precipitate.

Subsequently, the synthesized Fe_3_O_4_ MNPs were dispersed in 0.1 M (50 mL) Tetramethylammonium hydroxide (TMAOH) solution. Next, 0.1 mL Fe_3_O_4_/TMAOH solution and 100 mL DI water were stirred with sodium citrate (0.2 M, 3 mL) to replace surface hydroxide ions with citrate ions. Afterward, 1% (W/V) (0.5 mL) HAuCl4 solution and 0.2 M (0.2 mL) hydroxylamine hydrochloride (NH_2_OH·HCl) were iteratively added to the colloid to reduce the gold shells on the surface of Fe_3_O_4_ to form Fe_3_O_4_@Au core-shell MNPs^33^. The mixed colloid was continuously stirred during each iteration. In total 10 iterations were executed and every iteration took 20 minutes. Fe_3_O_4_@Au MNPs were obtained by centrifuging (6000 rpm, 30 min) and were then washed with DI water. The precipitate Fe_3_O_4_@Au MNPs were dispersed in ethanol (2 mL).

**Figure 6:**
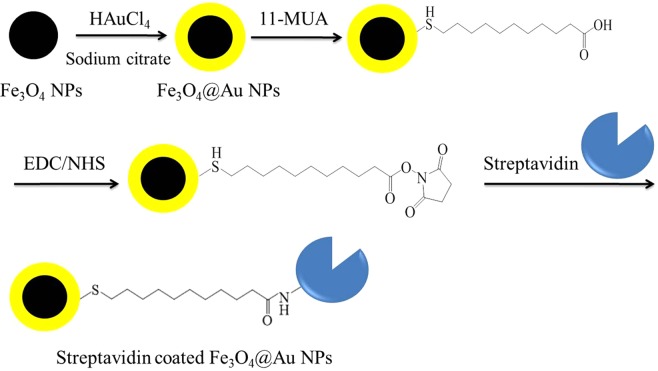
Flow chart of the synthesis process of streptavidin-coated Fe_3_O_4_@Au core-shell MNPs.

Surface modification was needed to bind STA onto the gold surface^34^. 11-mercaptoundecanoic acid (11-MUA) can be self-assembled on the gold surface of Fe_3_O_4_@Au MNPs and provides a carboxyl group for bioconjugation. Fe_3_O_4_@Au nanoparticles were added with 11-MUA (20 mM, 200 μL) and then continuously sonicated for 20 hours. We then centrifuged (12000 rpm, 10 min) the colloid and washed the precipitate with the ethanol. Afterwards, the precipitate was dissolved into PBS (0.001 M, 1 mL, pH 7.4). To activate the carboxyl groups on the gold surface prior to covalent coupling, N-(3-dimethylaminopropyl)-N’-ethylcarbodiimide hydrochloride (EDC) (20 mM, 200 μL) and n-hydroxysuccinimide (NHS) (40 mM, 200 μL) were added and continuously sonicated for 2 hours. Then the STA (1 mg/1 mL, 50 μL) was added and the mixed solution was sonicated for 1 hour to form Fe_3_O_4_@Au-STA MNPs. EDC and NHS enable the bioconjugation of carboxyl groups on the gold surface with the amino groups of STA molecules to form the streptavidin-coated Fe_3_O_4_@Au core-shell MNPs (Fe_3_O_4_@Au-STA). Finally, a liquid phase reagent containing Fe_3_O_4_@Au-STA MNPs was produced. Figure 6 shows the synthesis processes of Fe_3_O_4_@Au-STA MNPs.

### The Faraday rotation measurement setup

The Faraday rotation measurement was performed using AC magnetic fields and lock-in technique^35,36^. The light source was a diode-pumped solid-state laser with a wavelength of 532 nm. The frequency of the AC magnetic field was set at 813 Hz of which the environment noise was relatively lower. More details of the Faraday rotation measurement can be found in^30^. The measurement samples were prepared in liquid phase and encapsulated in sample holders made of glass. X-ray diffraction (XRD) was performed using BRUKER D8 SSS diffractometer with CuKα radiation.
